# THE ROLE OF THE OBESOGENIC ENVIRONMENT AND PARENTAL LIFESTYLES IN INFANT FEEDING BEHAVIOR

**DOI:** 10.1590/1984-0462/;2019;37;3;00005

**Published:** 2019-05-16

**Authors:** Rafaela Ramos Dantas, Giselia Alves Pontes da Silva

**Affiliations:** a Universidade Federal de Pernambuco, Recife, PE, Brazil.

**Keywords:** Feeding behavior, Modalities, alimentary, Family relations, Obesity, Child, Comportamento alimentar, Modalidades alimentares, Relações familiares, Obesidade, Criança

## Abstract

**Objective:**

To identify the role of the obesogenic environment and parental lifestyles in infant feeding behavior.

**Data sources:**

The searches were performed in PubMed, Medline, Cochrane, Lilacs and Scielo databases, in Portuguese, English and Spanish**.** The descriptors used were found in the Medical Subject Headings and in the Descriptors in Health Sciences being these: Comportamento alimentar/Feeding Behavior/ Conducta Alimentaria; Crianças/Child/ Niño; Relações familiares/Family Relations/Relaciones Familiares; e Ecologia/ Ecology/ Ecología. These were combined by the Boolean operator AND.

**Data synthesis:**

Researchers consider that parents (orprimary caregivers) are responsible, in part, for the unhealthy eating behavior presented by children, and for them to change it is necessary to change the behavior of the family, ensuring the correct choice of food and the practice of physical activity. The family environment has a significant impact on the development of eating behavior, so adults should provide a good model of this behavior for children.

**Conclusions:**

It was verified through this review that, in order to maintain and develop a healthy eating behavior, it is necessary to reach different spheres of life of the individual - physical, social, psychological, family, cultural and mediatic environment.

## RESUMO

**Objetivo:**

Investigar a influência do ambiente obesogênico e dos estilos de vida parentais no comportamento alimentar infantil.

**Fonte de dados:**

Foram consultadas as bases de dados PubMed, Sistema Online de Busca e Análise de Literatura Médica (MEDLINE), Cochrane, Literatura Latino-Americana e do Caribe em Ciências da Saúde (Lilacs) e Scientific Electronic Library Online (SciELO) nos idiomas português, inglês e espanhol. Os descritores utilizados foram selecionados no Medical Subject Headings e no Descritores em Ciências da Saúde (DeCS): comportamento alimentar*/feeding behavior/conducta alimentaria*; crianças*/child/niño*; relações familiares*/family relations/relaciones familiares;* e ecologia*/ecology/ecología.* A pesquisa foi especificada utilizando-se o operador booleano AND.

**Síntese dos dados:**

A maioria dos estudos sugere que os pais (osprincipais cuidadores) são responsáveis, em parte, pelo comportamento alimentar não saudável apresentado pelos filhos, e para que esse comportamento se modifique é necessário mudar o comportamento da família, garantindo a escolha correta de alimentos acompanhada da prática de atividade física. O ambiente familiar mostra impacto significativo no desenvolvimento do comportamento alimentar, por isso os adultos devem fornecer um bom modelo desse comportamento para as crianças. É importante ressaltar a contribuição do ambiente físico, da cultura, da escola e da mídia no processo.

**Conclusões:**

Verificou-se nesta revisão que a formação do comportamento alimentar depende da interação de fatores relacionados à criança e aos cuidadores e sofre a ação de diversos fatores relacionados à vida do indivíduo - ambientes físico, social, familiar, cultural e midiático.

## INTRODUCTION

Feeding behavior characterizes the way people feed themselves. The behavioral responses associated with the act of eating interfere in the quality of life and, when inappropriate, favor the onset of some chronic-degenerative diseases.^1^ Children’s feeding behavior is initially determined by the family, and subsequently by psychosocial and cultural processes.^1^

For almost two decades, Davison and Birch^2^ proposed a conceptual model that sought to explain the formation of this behavior from the interaction of different factors, such as: the characteristics of the child, parental practices, and the environment where these are exercised. In this perspective, there are two other models with similar characteristics: one by Contento and Michela,^3^ which seeks to explain food-related choices by referring to physiological, cognitive, and environmental factors (including family determinants); and the ecological model,^4^ ^,^ ^5^ which analyzes the integration of multiple determinants - proximal and distal - in the genesis of infant feeding behavior.

Davison and Birch^4^ and Tabacchi et al.^5^ have developed ecological models based on the theory of ecological systems by Bronfenbrenner,^6^ which summarize environmental influences on behavior, including feeding behaviors, at specific stages of development.

The 6Cs model^7^

 integrates several aspects: culture and society and characteristics of cities, communities, the family and the child. According to this broad view, this review was based on a focus on the obesogenic environment, family relationship and the child’s feeding behavior. Therefore, this study aims to investigate the influence of the obesogenic environment and of parental lifestyles on infant feeding behavior.

## DATA SOURCE

A narrative literature review was carried out, using the databases PubMed, Medical Literature Analysis and Retrieval System Online (MEDLINE), Cochrane, Latin American and Caribbean Health Sciences Literature (Lilacs) and Scientific Electronic Library Online (SciELO). he descriptors used were found in Medical Subject Headings and in Descriptors in Health Sciences (DeCS), namely: *comportamento alimentar*/feeding behavior/*conducta alimentaria*; *crianças*/child/*niño*; *relações familiares*/family relations/*relaciones familiares*; and *ecologia*/ecology/*ecología*. These were combined by the Boolean operator AND.

Review articles and original articles related to the influence of the obesogenic environment, parental lifestyles and other determinants of infant feeding behavior were selected. After reading the abstract, those articles whose object of study was the theme proposed in this review and that, according to the authors’ evaluation, were considered well-structured from a methodological point of view, were read in full. Studies that did not meet the criteria were excluded, even if their titles were suggestive to the theme.

## DATA SYNTHESIS

### Feeding behavior: an ecological look

Bronfenbrenner’s (bio)ecological human development theory (BHDT) highlights the importance and influence of the environment on human development. The author argues that development is a process that involves stabilities and changes, related to the biopsychological characteristics of individuals and of the environment, and that occurs throughout life and extends over generations.^6^

Over time, the main concepts have been reformulated, and the theory went on to explain development considering four interrelated aspects: the person, the process, the context and the time (PPCT model).^7^ It also proposes the construct of *proximal processes*, understood as “particular forms of interaction between organism and environment, which operate over time and comprise the first mechanisms that produce human development”.^8^

Regarding the *person*, Bronfenbrenner^9^ recognized the relevance of biological and genetic factors in the individual’s development. That is, the human being is considered a biopsychological being and constantly interacts with its context, being the product of this interaction process.^10^

The *process* is understood as the main development mechanism^10^ and concerns the interactions that take place between the individual and other people, as well as the relationships with the symbols and objects present in their environment. These forms of interaction, considered as proximal processes, would be the engines of development. They would occur according to the particular characteristics of the individual and of the context, both spatial and temporal,^10^ such as playing individually or in groups and learning new skills. Suchactivities are like gears of development, for it is through the form of engagement in these tasks and interactions thatthe individual becomes capable of giving meaning to their world, thereby transforming it.^10^

The *context* is represented by any fact or condition that can influence or be influenced by the developing being.^11^ Finally,there is *time*. Bronfenbrenner^12^ came across the issue of time and its influence on human development and created the concept of *chronosystems* by establishing a research model that examines the influence and changes that occur in the environment over time.^12^

In addition, the author shows that these influences differ between people in terms of extent and the type of consequences.^6^ Aiming at a better understanding of the possible interactions, he proposes the existence of four socially organized subsystems, which would guide the process: microsystem, mesosystem, exosystem and macrosystem.^11^

**Figure 1:**
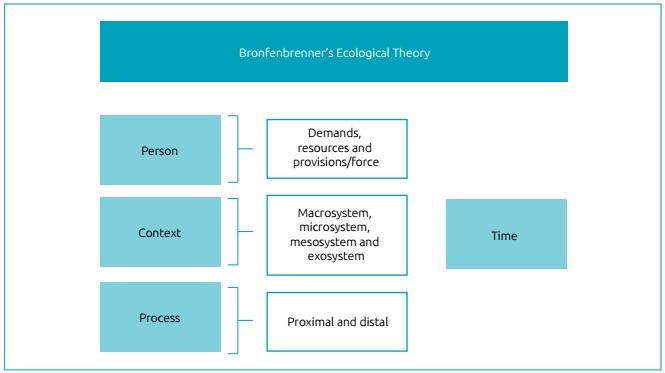
Schematic presentation of the topics and subtopics of the ecological theory according to Urie Bronfenbrenner.

The *macrosystem* has a broader scope and is composed of all other systems. *Microsystems* are like primary development contexts. That is, the closest environments in which roles, face-to-face interactions and activities occur, in which the individual observes and is influenced by groups or more experienced people to perform activities,^11^ such as family, considered the first microsystem, and school.^13^ The *mesosystems* encompass two or more microsystems in which the developing person is inserted. That is, it is the link between the school and the religious institution, between the workplace and the family, between the neighbors and the day care center.^11^ The *exosystem* is represented by environments in which the person is not physically present, but the decisions taken there influence their development.^11^ For example, the stressful work environment of parents may result in poorer quality care for their children in the home environment (having a negative influence) (Figure 1).^11^

The ecological theory seeks to explain the relationships (processes) that are established during personal or social growth, over the course of the individual’s history (time) in a given context. The PPCT model allows to analyze the risk and protection mechanisms present in the environment and in the family environment related to feeding behaviors, making this complex phenomenon easier to understand.

### Feeding behavior: a process

The formation of *feeding behavior* begins in childhood in the first months of life and, over time, is the result of the interaction between genetic and environmental factors.^14^ Breastfeeding,the introduction of complementary foods after six months of life, family eating habits and socioeconomic conditions play a key role in this formation.^15^ ^,^ ^16^

The strong family influence is mediated by parenting styles.^17^ Parental attitudes related to the dietary regulation of their children characterize what is called *parental feeding control*, classified into three types: restrictive control, pressure to eat, and surveillance or discrete control.^17^

*Restrictive control* involves the exclusion of foods considered unhealthy and interferes with the amount consumed by the children, reflecting an excessive preoccupation of mothers or caregivers with food.^18^ For Garcia et al.,^19^ the children who present the most difficulties in regulating the caloric intake are those with more controlling mothers.

Parents should encourage their child to eat in moderation, leading them to associate the onset of a meal with the feeling of hunger and the end of the meal to the feeling of satiety.^19^ Saltzman et al.^20^ found that maternal compulsive behaviors are associated with increased energy intake and higher body mass index in children. That is, inappropriate maternal behavior reflects on the child’s feeding behavior.

The *pressure to eat* is characterized by the pressure from the parents for the child to choose healthier foods - fruits and vegetables -, or to eat everything on the plate.^21^ For Vandeweghe et al.,^22^ this leads to loss of sensitivity to internal signs of satiety and the child starts to use external signs (favorite food, smell of food) or emotions as signs of hunger or satiety. Consequently, the child relies on external stimuli to start, maintain, and finish their meal.^22^

In the case of *discrete control*, there are few studies. Tayloret al.^23^ observed that it was related to the high consumption of fruits and vegetables, which suggests that less controlling attitudes and more psychological support are associated with lower intake related to the child’s emotional state. In short, parental behavior can shape and/or alter their child’s feeding behavior.^24^

####  Parenting styles and feeding behavior 

Aspects related to parental responsibility (or lack thereof) identify the styles they adopt in their children’s social education and which reflect on their behavior.^24^ *Parentingstyles* are considered as a set of attitudes that form the emotional climate in which parental behaviors are translated.^25^ It also takes into account how parents deal with issues of power, hierarchy and emotional support regarding their children.

The concept of parental style according to Baumrind^26^ integrates behavioral and affective factors that involve child rearing based on the influence of parental authority on child development, reformulating the ancestral view of control, hitherto defined in terms of radicality and physical punishment. Three categories of parental style are established: authoritative or democratic, authoritarian and permissive. *Authoritative* parents value autonomy and the exchange of ideas and exercise firm control, but are responsive. The *authoritarian* style is one in which parents proceed with a high degree of control and expect obedience, make use of negative reinforcement and punitive measures.^25^ Finally, the *permissive style* is characterized by the affectivity of the parents in relation to the children and by the lack of control over their behaviors. Parents of the latter category, according to Baurimd,^26^ (almost always) allow the child to exercise actions based on impulsive and momentary desires.

In the early 1980s, Maccoby and Martin,^27^ based on Baumrind’s theoretical model, proposed two fundamental dimensions of parental educational practices, called demandingness, when parental behaviors seek to control their children’s behavior in some way, establishing limits and rules; and responsiveness, when the understanding behaviors that parents have toward their children seek, through emotional support and dialogue, to contribute to the development of autonomy and self-assertion from early childhood.^27^

These authors suggested four parental styles: authoritative, authoritarian, indulgent, and negligent. Parents with high responsiveness and demandingness are classified as *authoritative*, establishing rules that are consistently emphasized. Correct attitudes are gratified, and wrong ones are corrected. Communication is clear and open, based on mutual respect, and “rules” are imposed in an inductive way. They are affectionate in interaction, responsive to needs, and often solicit their children’s opinions.^26^ ^,^ ^27^

The sum of a high level of control and little responsiveness results in the *authoritarian* style, in which parents are rigid, radical and autocratic; they impose a high degree of demandingness, establishing strict rules, regardless of the child’s participation. Usually, they emphasize obedience through respect for authority and order. Punishment is the form of control, and do not value dialogue or autonomy; they also show low responsiveness to the questions that the child may have.^26^

The *indulgent* style is the combination of a low level of control and a high level of responsiveness. These parents do not set rules or limits for the child; they do not require responsibility or maturity. They are affectionate, communicative and receptive, with a tendency to fulfill any of the child’s needs. Tolerance is exercised excessively, allowing the child to control themselves.^26^

The *negligent* style is the result of low levels of control and responsiveness. These parents are neither caring nor demanding of children. They have little social interactivity and do not watch over the behavior of their children. They respond only to basic needs, which generates some distance. They are concerned and focused on their own interests.^26^

**Figure 2:**
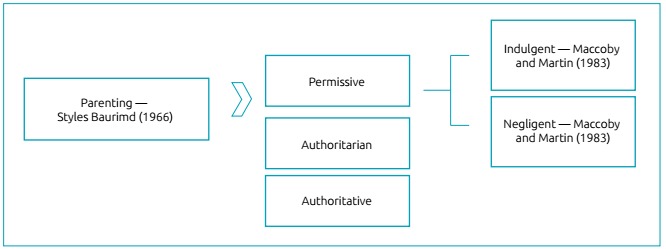
Parental styles, according to Baumrind,^26^ and later modified by Maccoby and Martin.^27^

The difference between Baumrind’s proposal^26^ and Maccoby’s and Martin’s new proposal^27^ is the dismemberment of the permissive style classification into two other styles: the indulgent and the negligent (Figure 2).

Other studies with older children and adolescents have shown that the paternal parenting style influences health behaviors and the child’s well-being.^28^ ^,^ ^29^ The authors concluded that fathers, not just mothers, are important and should be included in research on the influence of parental styles and various situations of health risk to the child, since most studies have focused exclusively on the role of mothers and on the fact that the father’s affectionate and firm style is associated with better child behavior.^29^ ^,^ ^30^

Since the development of unhealthy feeding behaviors and low levels of physical activity are associated with harmful outcomes and, in the early years, these behaviors occur to a large extent in the family unit, it is relevant to analyze the influence of parents in this formation. The family unit is the primary context for providing essential care, resources and opportunities for a healthy development.^30^

####  The family environment and cultural influences 

The family is considered the first and foremost agent of socialization, transferring and/or shaping behaviors and lifestyles to children, often through their own practices.^31^ It is in the family environment that this learning begins, where the first feeding experiences happen.^21^ ^,^ ^32^

Some researchers believe that parents (or primary caregivers) are responsible, in part, for the unhealthy feeding behaviors presented by children, and for these behaviors to change, the family behaviors need to change, ensuring the correct choice of foods accompanied by the practice of physical activity.^32^ ^,^ ^33^ Although parents are not the only food providers (there are also, among others, the school environment and day care), they play a key role, especially in the early years of the child’s life.^34^ ^,^ ^35^

The way we feed ourselves, our preferences and rejections to particular foods are, during childhood, strongly conditioned by the family context and often reflect the feeding behaviors of the community.^36^ ^,^ ^37^ Therefore, when interventions involve the family in studies on the prevention/treatment of various diseases, better success rates are observed than when using conventional treatments, which include feeding and physical activity counseling aimed only at the child.^38^

Thus, the family environment has a significant impact on the development of feeding behaviors. Thus, adults should provide children with a good model of this behavior.^39^ Thefamily must be the first ally in preventive or interventionist actions, because dietary patterns that can last a lifetime also originate in the family;^40^ and these patterns suffer cultural influences.

In general, over the last 60 years, social, economic and technological changes have occurred that have altered the lifestyles of populations in different countries. In addition to this, there were changes in feeding behaviors, influenced by environmental changes (micro and macroenvironments),^41^ most of which contribute to establishing an obesogenic environment, directly or indirectly influencing the adoption of behaviors that will affect health, either positively or negatively.^42^

### Feeding behavior: influence of the obesogenic environment

In 2011, Harrison et al. presented an ecological model that addresses not only hereditary but also environmental influences on childhood obesity, termed 6Cs. The 6Cs model addresses six spheres: cell, child, family, community, country, and culture. The *cell* represents genetic vulnerabilities and other biological factors; the *child*, personal and behavioral characteristics; the *family* is represented by family characteristics, such as parental dynamics and household rituals; the *community* includes factors related to the child’s social world of outside the household; and the *country* indicates state and national institutions that seek to influence the behaviors of citizens by means of recommendations. Finally, *culture* involves cultural norms, myths, and prejudices, which influence political decisions regarding food, exercise, health, and the body.^7^

According to Swinburg et al.,^43^ the obesogenic environment is characterized as the presence of opportunities and environmental conditions that favor the installation of obesity. From a dietary standpoint, it can be conceptualized as a *space* in which beliefs and behaviors are associated with the availability of processed, energetically dense, nutrient-poor foods, and the absence of foods rich in fiber, vitamins, and minerals.^44^ It covers physical, economic and cultural factors related to food and physical activity.^43^

**Figure 3:**
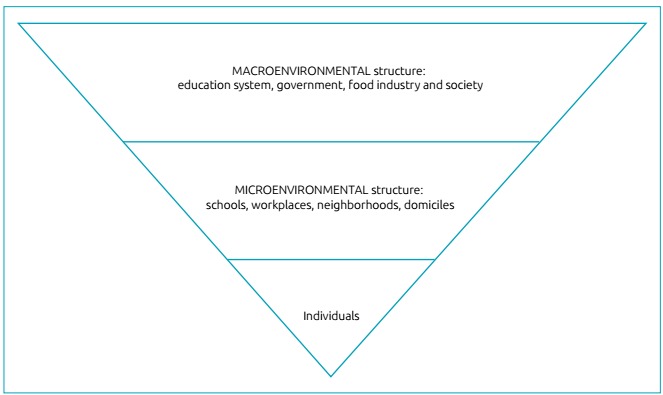
Schematic presentation of environmental influences suffered by individuals, according to Swinburn et al.^43^.

The individual lives in microenvironments (home, school, workplace, neighborhood), which, in turn, are influenced by the macroenvironment (education system, government, food industry).^43^ Microenvironmental influences have been the most addressed in the literature, perhaps for being easy to study (Figure 3).

The environment in which we live is considered to be obese because it leads us to practice unhealthy behaviors.^45^ One of the analyzed components is the physical area in which the individual resides, that is, if the surrounding areas have establishments where they can acquire healthy foods and conditions for the practice of physical activity. It also includes the availability of processed and ultraprocessed foods^46^ and leisure facilities^44^ at home.

Pearson et al.^46^ avaliaram o ambiente em que as crianças estavam inseridas e verificaram que aquelas cujos pais possuíam mais acesso à compra de alimentos saudáveis apresentavam práticas alimentares mais adequadas.

A study by Jaime et al.^47^ in São Paulo analyzed the relationship between the surrounding environment (food trade) and eating behaviors, physical activity and overweight. Theauthors showed that a greater number of places selling fruit were associated with better feeding behavior and the intake of nutrient rich foods.

Thus, the existence of environments considered as obesogenic represents one of the greatest difficulties for the maintenance of a healthy lifestyle. For this reason, it is key to know the components of this environment, as well as the evaluation of the role and interaction of the factors that compose it.^48^ In this context, the influence of the various media vehicles in the formation of children’s behaviors must also be considered, a fact that has been valued in recent years.

####  The role of media 

The media can be considered as an important agent influencing feeding behaviors, since it establishes food consumption patterns from very early on by the way it diffuses information. The visual appeal of advertisements related to food products intended for children should be seen as a public health problem.^49^

Thimming et al.^50^ explain that the choice of foods low in nutrition and high in caloric density is stimulated by the habit of watching television. Because of the immaturity of children in critical thinking and decision making, they are vulnerable to commercial appeals and bombarded with advertising aimed specifically at children.

Benetti et al.^13^ conducted a study showing the association between excessive child exposure to television and the internet and unhealthy living practices. Shi and Mao^51^ conducted a study in California (United States) and found a significant association between the time a child watches television and their feeding behavior.

One of the reasons why television and other media influence infant feeding behavior is that food is the most advertised category of products in children’s TV programming, and it has been established that exposure to food advertisements effectively promotes the consumption of the products advertised.^52^

Current media strategies are widely used techniques by advertising companies to stimulate the consumption of their products. There is a strong media investment in fast food, which is high in calories, and carbonated beverages (water, carbon dioxide and some type of syrup that gives the drink its color and taste), sugar-rich cereals and snacks, as well as foods rich in fats, sugar and sodium that are poor in nutrients.^53^

Faced with the increasing proportions of global prevalence of chronic noncommunicable diseases, many researchers have suggested that food media contributes to an obesogenic environment, making healthy choices more difficult, especially for children.^14^ In addition, television also contributes to sedentarism, diverting the individual from activities that could help in burning excessive calories contained in unbalanced diets.^54^

## CONCLUSION

This review has shown that for the maintenance and development of healthy feeding behaviors, it is necessary to reach different aspects of the individual’s life - physical, social, psychological, family, cultural and mediatic environments. In relation to the young age groups, it is essential for health professionals to understand all the factors involved, since it is possible to promote actions aimed at providing good health conditions to the child even under unfavorable external conditions.

Contrary to a deterministic view, a number of empirical studies have shown that even if a child is growing up in an obesogenic environment with authoritarian or negligent parents, with an unfavorable regional culture and feeding on noxious media, these factors can be minimized by interventions at theindividual, familiar and collective levels, contemplating the different aspects of the problem.
